# Phase synchrony facilitates binding and segmentation of natural images in a coupled neural oscillator network

**DOI:** 10.3389/fncom.2013.00195

**Published:** 2014-01-27

**Authors:** Holger Finger, Peter König

**Affiliations:** ^1^Institute of Cognitive Science, University of OsnabrückOsnabrück, Germany; ^2^Institute of Neurophysiology and Pathophysiology, University Medical Center Hamburg-EppendorfHamburg, Germany

**Keywords:** oscillation, binding, synchronization, normative model, unsupervised learning, scene segmentation, object label, natural image statistics

## Abstract

Synchronization has been suggested as a mechanism of binding distributed feature representations facilitating segmentation of visual stimuli. Here we investigate this concept based on unsupervised learning using natural visual stimuli. We simulate dual-variable neural oscillators with separate activation and phase variables. The binding of a set of neurons is coded by synchronized phase variables. The network of tangential synchronizing connections learned from the induced activations exhibits small-world properties and allows binding even over larger distances. We evaluate the resulting dynamic phase maps using segmentation masks labeled by human experts. Our simulation results show a continuously increasing phase synchrony between neurons within the labeled segmentation masks. The evaluation of the network dynamics shows that the synchrony between network nodes establishes a relational coding of the natural image inputs. This demonstrates that the concept of binding by synchrony is applicable in the context of unsupervised learning using natural visual stimuli.

## 1. Introduction

One of the central questions in neuroscience is how information about a given stimulus is processed in a distributed network of neurons such that it is perceived not only as a collection of unrelated features but as a unified single object. The concept of binding by synchrony has been proposed as a mechanism to coordinate the spatially distributed information processing in the cortex (Milner, 1974; Von Der Malsburg, 1981). Experiments in cat visual cortex have confirmed that inter-columnar synchronization indeed corresponds to a relational code that reflects global stimulus attributes (Gray et al., 1989; Singer, 1999; Engel and Singer, 2001). However, the physiological recordings in these early studies were based on the presentation of artificially designed stimuli. In a more recent study Onat et al. (2013) showed in experiments that long-range interactions in the visual cortex are compatible with Gestalt laws. This suggests that the concept of binding by synchrony is also feasible in the case of natural visual stimuli. It is still the center of a heated debate to what extend synchronized activity represents a neural code of binding and segmentation. Especially, how the neural system can learn this relational coding when it is exposed to new stimuli is still an open question. The most prominent possibility is that tangential cortico-cortical connections in the visual cortex lead to synchronized activity that implements Gestalt laws. Löwel and Singer (1992) showed in cats with artificially induced strabismus that selective stabilization of tangential connections occurs between cells that exhibit correlated activity induced by visual experience. Furthermore, König et al. (1993) found that the synchronization of cortical activity is impaired in these cats with artificial strabismus. These findings indicate that there is an important interplay between unsupervised learning of tangential connections on behavioral time scales and their role in synchronization phenomena on fast time scales.

The physiological experiments on binding by synchrony have been accompanied by theoretical studies early on. Sompolinsky et al. (1990) investigated how a model of coupled neural oscillators is able to process global stimulus properties in synchronization patterns using abstractly defined neuronal activation levels and predefined coupling strengths for the simulated network. These simulation results showed that the coupling of neural oscillators provides a viable mechanism implementing a coding of perceptual grouping. Such previous work includes studies ranging from networks build out of very simple elements to detailed simulations containing many compartments per unit.

To investigate the functional role of synchronization and its relation to coding, it is important to choose the right level of abstraction in the model. A simplification from detailed spiking neuron models to coupled phase oscillator models allows us to analyze neuronal synchronization in a broader context of a normative model involving unsupervised learning from natural stimuli. A review of these coupled neural oscillator models was done by Sturm and König (2001), where the authors show the derivation of simplified phase update equations from biologically measurable phase response curves. The simplifications in coupled phase oscillators are based on the assumption that neurons are close to their oscillatory limit-cycle and that a change in the phase of the neuronal inputs induces only a small perturbation to the neuronal phase. The phase update equation in our model is based on the Kuramoto model of coupled phase oscillators (Kuramoto, 1984) in the sense that our model also assumes a very simple sinusoidal phase interaction function. This approximation of the phase interaction by a sinusoidal function allows us to use mathematical simplifications in the simulation of the model.

Very similar to the work of Sompolinsky et al. (1990), we extend the standard formulation of the Kuramoto model with a second variable per neuron to encode the activation of the oscillators. Therefore, in our model the state of a neuron is represented by 2 degrees of freedom, which are separated into activation and phase variables. This discrimination between coding of receptive field features by activation and coding of relationships by phase is a biologically motivated segregation of their different functional roles. Maye and Werning (2007) specifically compare the synchronization properties of these coupled phase oscillator models with mean-field oscillator models based on the Wilson-Cowan model (Wilson and Cowan, 1972). They state that the simplified coupled phase oscillators allow decoupling the simulation time constants of fast oscillatory time scales from slow rate coding time scales. Another advantage is an easier analysis of the synchronization patterns, because the direct encoding of the phase variables means that all contextual relationships are coded at the same time. Consequently, we use the dual variable phase model, because it is suitable to answer fundamental questions about the interactions between synchronization phenomena and contextual coding in neural systems.

In contrast to these phase oscillator models, most recent work on segmentation in networks of coupled neural oscillators is based on the so called “local excitatory global inhibitory oscillator network” (LEGION) or similar variants of this model, which was first proposed by Wang and Terman (1997). In LEGION the dynamics of each oscillatory period of individual units is simulated in detail by time-varying variables describing the internal states of each neuron. In contrast, in our model the oscillatory period is not simulated, but represented only implicitly in the phase variables. Nonetheless, several aspects which we analyze in this work were previously also investiged in LEGION. Namely, similar to Li and Li (2011) we use a small-world topology, to reduce the computational cost while still allowing binding by synchrony over large distances. We also use parallel computations to speed up the simulations, which was also previously done in LEGION by Bauer et al. (2012).

The above-mentioned previous theoretical studies mostly investigated the processing of artificial stimuli in close analogy to the physiological experiments. These stimuli are heavily dominated by artificial geometric patterns as bars and gratings. However, the concept of binding by synchrony makes much more general claims about grouping of sensory representations of natural stimuli. By now a fair number of databases with images considered to be natural is available. However, a problem with generic natural stimuli is that segmentation is not only difficult, but no general ground truth is available. The LabelMe database (Russell et al., 2008) is rather unique, as it contains a large collection of images together with human labeled annotations of image segments. In theoretical studies these labels may serve as a ground truth to evaluate how the relative phases between neurons are coding relational structures on natural stimuli.

The processing of natural stimuli in neural systems can be described as a normative approach in which the representation of the input is learned by an optimization of computational principles (Einhäuser and König, 2010). It has been successfully employed in modeling receptive field properties of simple and complex cells in primary visual cortex. Furthermore, response properties of neurons in higher areas and other modalities have been suggested to follow similar rules. This approach might be extended to include the computational principles that underlie tangential interactions that directly influence synchronization phenomena. This might answer the question whether the concept of binding by synchrony can work in principle with unsupervised learning and natural stimuli.

In this study we investigate whether the concept of binding by synchrony, as has been investigated using abstract stimuli, is viable for natural stimuli. The most important novelty of our approach is the combination of these different concepts described above into one single simulation model to allow the investigation of their interplay: Specifically, we combine normative model approaches of unsupervised learning from natural stimuli with the concept of binding by synchrony in a network of coupled phase oscillators. Importantly, the data driven approach, that utilizes general principles, minimizes the number of heuristics and free parameters. We present large-scale simulations of neural networks encoding real-world image scenes. In the first stage of our algorithm forward projections generate activation levels of neurons corresponding to the primary visual cortex. In the second stage these activation levels are used in a simulation of tangential coupled phase oscillators. We present results with forward projections based on designed Gabor filters that are a good approximation of receptive fields in the primary visual cortex. To allow later canonical generalization in higher network layers, we also present results with forward projections learned in a normative model approach with a sparse autoencoder using natural image statistics. In addition to these learned forward weights, the structural connectivity of the phase simulations is also learned unsupervised using the correlated activity induced by natural stimuli. Performance of the network is tested using images taken from the LabelMe database. Thereby we can investigate how synchronization phenomena might be utilized in sensory cortical areas to bind different attributes of the same stimulus and how it might be exploited for scene segmentation.

## 2. Materials and methods

**Figure 1:**
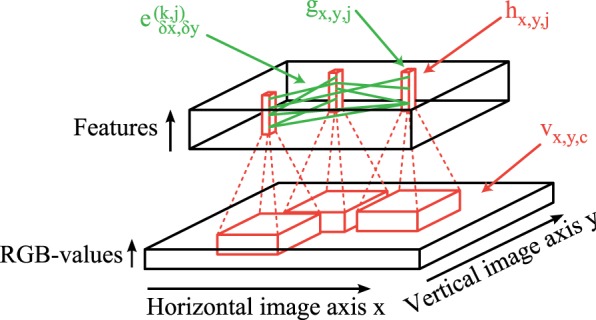
**Network structure**. Feedforward convolutional filters (red) are applied on the input image *v*_*x,y,c*_ (bottom) to generate the activation levels *h*_*x,y,j*_ of feature columns (red blocks in the top). These activations *h*_*x,y,j*_ are then transformed to activations of oscillators *g*_*x,y,j*_ using simple local regularization steps. The intralayer connections *e*^*k,j*^_δ*x*,δ*y*_ (green) simulate these coupled phase oscillators which synchronize or desynchronize image features.

The overall network architecture of our simulation model consists of two main parts: (1) Feedforward convolutional filters (red lines in Figure 1) are used to generate the activation levels for neurons in a layer corresponding to the primary visual cortex. On top of each pixel is a column of neurons which encode different features of a local patch in the input image (black bottom cuboid in Figure 1). Each feature type is described by a weight matrix which is applied using a 2-dimensional-convolutional operation on each rgb-color-channel of the input image. (2) The obtained activation levels in this 3-dimensional structure (black top cuboid in Figure 1) are subsequently used to simulate sparse connections (green lines in Figure 1) between coupled phase oscillators.

We start with the description of the stimulus material (section 2.1). This is followed by the description of the coupled phase oscillator model (section 2.2) and the sampling mechanism generating the horizontal sparse connections (section 2.3). Afterwards we describe the underlying mechanism of the feedforward generation of activation levels (section 2.4).

### 2.1. Natural stimulus material

As stimulus material in our simulations we use images of suburban scenes from the LabelMe database (Russell et al., 2008). Due to computational time constrains we have to restrict the evaluations to a small subset of all available images in the database. In addition, the database is not fixed but new images and segmentation masks are often added. We use only the first 50 images in the folder *05june05_static_street_boston* so that we have a consistent and fixed dataset of well defined images.

These images have initially a resolution of 2560×1920 pixels. We first resize the images to 400×300 pixels to further reduce the computation time of the simulations. Subsequently we subtract the mean pixel values and apply a smoothed zero-phase (ZCA) whitening transformation (Bell and Sejnowski, 1997). For an input image *X* the whitened pixel values are given by *X*_ZCA_ = *UDU^T^X*, where *U* is a matrix containing the eigenvectors of the covariance matrix of the image statistics and *D* is a diagonal matrix with diagonal elements $\frac{1}{\sqrt{\lambda_{i}+0.1}}$ where λ_*i*_ are the corresponding eigenvalues. This transformation applies local center-surround whitening filters that decrease the correlations in the input images. We implement this whitening transformation using a convolutional image filter.

The images in the LabelMe database come along with human labeled segmentation masks. These segmentation masks correspond to objects that are perceived as a unique concept with an associated abstract label like “tree,” “car” or “house.” We use these supervised segmentation masks for later evaluations of binding in the simulated phase maps. Please note that in our network simulations this segmentation information is not used at any moment in time. Instead, the network connectivity is based solely on unsupervised learning using the statistics of neuronal activations.

### 2.2. Coupled phase oscillator model

Our network of coupled phase oscillators is based on the oscillator model described by Sompolinsky et al. (1990). In the following, we use the same motivational derivation of the phase update equations. We model the probability of firing *P*_*x,y,k*_(*t*) per unit time of a neuron at image position (*x*, *y*) encoding feature type *k* at time *t* by an isochronous oscillator. In our simulations we represent the state of the neuronal oscillators by seperated activation variables *g*_*x,y,k*_ and phase variables Φ_*x,y,k*_. These two variables are linked to the biological interpretation of firing probability by the equation

(1) $$P_{x,y,k}(t)=g_{x,y,k}\left(1+\lambda \cdot \cos(\Phi_{x,y,k}(t))\right),$$

where the parameter 0 < λ < 1 controls the relative strength of the temporal oscillation in relation to the overall firing probability of the neuron. The phase progression is a periodic function Φ_*x,y,k*_(*t*) = Φ_*x,y,k*_(*t* + 2π). In our work, the calculation of the activation levels *g*_*x,y,k*_ significantly differs from the simple artificial tuning curves used in Sompolinsky et al. (1990). A detailed description of how these activation levels are obtained will be presented in section 2.4. The activation levels *g*_*x,y,k*_ are normalized by dividing by the local sum of all activation levels at each image position such that ∑_*k*_*g*_*x,y,k*_ = 1∀*x*, *y* ∈ ℤ. In the simulations presented in this work the activation levels of each neuron are only computed once from the input image using feedforward projections (red lines in Figure 1) and are then kept constant during the simulation of the phase model. This simplification of constant activation levels is based on the assumption that the stimulus presentaion on behavioral timescales (≈ seconds) remains constant during the phase synchronization which happens at very fast timescales (i.e., gamma frequency ≈ 40 Hz). Another argument to support this assumption is that the visual cortex seems to operate in a regime of self-sustained activity (Stimberg et al., 2009) and therefore we can assume constant activation levels during the phase simulation.

After these activation levels *V* are computed, we simulate the horizontal coupling between the phase oscillators. The phase connections in our network are described by a weighted graph *G* = (*H*, *E*) where the neurons *g*_*x,y,j*_ ∈ *H* are the vertices organized in a three dimensional block (Figure 1). An edge *e*^(*j,k*)^_δ*x*,δ*y*_ ∈ *E* describes synchronizing (positive) or desynchronizing (negative) connections from neurons *g*_*x,y,j*_ to neurons *g*_*x* + δ*x,y* + δ*y,k*_. The phase of each neuron is then modeled according to a differential equation describing weakly coupled phase oscillators (Kuramoto, 1984)

(2) $$\begin{array}{l} \frac{d\Phi_{x,y,k}(t)}{dt}=\omega -\frac{1}{\tau }\sum_{e_{\delta x,\delta y}^{\left(j,k\right)}\in E}g_{x,y,k}\cdot e_{\delta x,\delta y}^{\left(j,k\right)}\cdot g_{x-\delta x,y-\delta y,j}\cdot \\ \;\sin\text{​}\left(\Phi_{x,y,k}(t)-\Phi_{x-\delta x,y-\delta y,j}(t)\right), \end{array}$$

where τ is the time scale of the phase interactions and ω is the natural frequency of the modeled neural oscillations. We assume that all neurons have the same intrinsic natural frequency ω and the interaction strength *g*_*x,y,k*_ · *e*^(*j,k*)^_δ*x*,δ*y*_ · *g*_*x* − δ*x,y* − δ*y,j*_ is proportional to the activation levels of the pre- and post-sysnaptic neurons. Note that our model is in contrast to the more common formulation of the Kuramoto model with heterogeneous frequencies and fixed homogenous all-to-all interaction strengths.

A major difference to the phase update equation used in Sompolinsky et al. (1990) is that we neglect the noise term in the differential equation of each oscillator. The noise term in Sompolinsky et al. (1990) is used as the primary source of desynchronization in the network. In contrast, in our work, we use a normative model to learn not only synchronizing but also desynchronizing connections (see section 2.3). For an easier analysis and interpretation of the results, it is advantageous to have only a single source for the desynchronization in the network. Therefore, we decided to use a deterministic phase model, although it was previously shown that noise is an important factor to control the network coherence. In addition to a simpler interpretation it reduces the number of model parameters and is also more compatible to further applications of gradient descent learning to change the strength of the phase interactions.

We can further simplify the equation by using the fact that we model isochronous oscillators with homogeneous frequencies. In Equation 2 all phase variables Φ_*x,y,k*_(*t*) have a constant phase progression with frequency ω. We can use a simple transformation to a new variable, which represents only the phase offsets between neurons:

(3) $$\varphi_{x,y,k}(t)=\Phi_{x,y,k}(t)-\omega t.$$

This new phase variable φ_*x,y,k*_(*t*) describes the relative phase of neuron *k* to the global fixed network oscillation with frequency ω. Substitution into the equation above leads to a simplified phase update equation

(4) $$\begin{array}{l} \frac{d\varphi_{x,y,k}(t)}{dt}=-\frac{1}{\tau }\sum_{e_{\delta x,\delta y}^{\left(j,k\right)}\in E}g_{x,y,k}\cdot e_{\delta x,\delta y}^{\left(j,k\right)}\cdot g_{x-\delta x,y-\delta y,j}\cdot \\ \;\sin\text{​}\left(\varphi_{x,y,k}(t)-\varphi_{x-\delta x,y-\delta y,j}(t)\right). \end{array}$$

In this equation it can be seen that the timescale τ of the phase interaction strength is decoupled from the oscillatory timescale 1/ω. Please also note, that a change of the parameter τ would not qualitatively change the results of our simulations. Instead it would just linearly change the units of the time axes. Therefore, we show the simulation results with the time axis measured in iterations, which could be linearly scaled to arbitrary time units to best fit to different biological measurements.

This phase update equation is used in our simulations to model the horizontal connections in the network. It allows directly specifying synchronizing interactions from neuron *g*_*x,y,j*_ to neuron *g*_*x* + δ*x,y* + δ*y,k*_ with a positive connection weight *e*^(*j,k*)^_δ*x*,δ*y*_ and desynchronizing interactions with a negative weight respectively. We simulate these coupled differential equations using a 4th-order Runge-Kutta method.

### 2.3. Horizontal interaction strengths

We use correlation statistics of the induced activation levels to set the intralayer connection strengths similar to a simple Hebbian learning rule. We write ρ^(*k,m*)^_*x,y*_ to denote the Pearson cross-correlation between the activations of feature type *k* at image position ($\tilde{x}$,$\tilde{y}$) and the activations of feature type *m* at the shifted image position ($\tilde{x}$ + *x*, $\tilde{y}$ + *y*). Each correlation value in this tensor is calculated from the correlation statistics over approximately 1 million network activations induced by 50 natural images and presented at 236 × 86 image positions.

These horizontal connections make up the coupling between the neural oscillators. Instead of full connectivity, we use stochastically sampled sparse directed connections from the correlation matrix. To exclude noise in the correlation matrix, we use the Benjamini-Hochberg-Yekutieli procedure (Benjamini and Yekutieli, 2001) under arbitrary dependence assumptions with a false-discovery rate of 0.05.

The probability of a positive (+1) or a negative connection (−1) in the connectivity graph *G* = (*H, E*) is then given by

(5) $$P\left(e_{x,y}^{\left(j,k\right)}=\pm 1\right)=\eta_{\pm }\cdot \frac{\max\left(0,\pm \rho_{x,y}^{\left(j,k\right)}\right)}{\sum_{\tilde{x},\tilde{y},m}\max\left(0,\pm \rho_{\tilde{x},\tilde{y}}^{\left(m,k\right)}\right)},$$

where η_+_ specifies the total number of afferent synchronizing connections and η_−_ the total number of afferent desynchronizing connections per neuron. Therefore, synchronizing connections exist only between naturally correlated features and desynchronizing connections between anti-correlated features.

We sample this sparse tangential connection pattern such that it is invariant to spatial shift transformations. The convolutional structure of the forward projections leads to activation and phase variables that are stored in a 3-dimensional block (top of Figure 1) with two dimensions given by the spatial extend of the image and one feature dimension. This convolutional structure can be exploited for the sparse horizontal connections to significantly speed up the computation. Therefore, we specify the properties of the coupled oscillator connections only for a generic feature column. These connections are then applied at each image position. Specifically, in our implementation each sampled tangential connection is specified by 5 variables: the horizontal and vertical connection length in image directions and the indices of the afferent and efferent feature maps and the connection weight. This has the advantage that the phase update equation can be implemented as a vectorized convolutional operation although the connection pattern is highly sparse.

### 2.4. Feedforward connectivity

We compare the binding and segmentation performance of the coupled neural oscillator model using two different ways to generate the activation levels for the neurons. We first describe hand-crafted feedforward Gabor weights (section 2.4.1) and then the unsupervised learning of receptive fields using a convolutional autoencoder (section 2.4.2). Finally, activation functions are presented to further regularize the resulting feature representations (section 2.4.3).

#### 2.4.1. Gabor filters

For reference we use a set of Gabor filters with specified orientation, frequency and color tuning to generate the activation levels for the phase simulation. Thereby we can analyze the phase oscillator network based on a regularly defined set of features that can be parameterized.

We generate linear convolutional weights (marked in red in Figure 1) using an approximate Gaussian derivative model, which was shown to be a good fit for the receptive fields of simple cells in the primate visual cortex (Young, 1987). We use only non-directional three-lobe monophasic receptive fields (Young and Lesperance, 2001) to reduce our model parameters. We implement the Gaussian derivative model using difference-of-offset-Gaussians with a slightly larger center compared to surround to code color offsets. The receptive fields that are used in our simulations have a size of 12x12 pixels and are defined by

(6) $$W_{x,y}=g_{2\sigma }(y)\cdot \left(-5\cdot g_{\sigma }(x+\sigma )+10.1\cdot g_{\sigma }(x)-5\cdot g_{\sigma }(x-\sigma )\right),$$

where *g*_σ_(*x*) is a one dimensional Gaussian distribution with standard deviations σ = 1.5 pixels (or *g*_2σ_(*y*) with standard deviation of 2σ = 3 pixels) and the coordinates *x* and *y* are rotated giving a total of 8 orientations in steps of 22.5°. The convolutional filters are applied to the images with a stride of 2 pixels in both image dimensions and are followed by a sigmoidal activation function to scale the values to a reasonable interval between 0 and 1. We apply each orientation filter separately to all color channels (red, green, blue). Furthermore, we add features for the complementary color channels similar to the on-off discrimination in the visual pathway from the retina to the visual cortex. The direct linear dependency between these pairs of opponent-color channels is removed later with additional activation functions described in section 2.4.3. In summary, we have a total of 48 convolutional feature channels per image position: 8x orientations, 3x rgb-color channels, 2x opponent-color channels. This overcomplete neural representation of the input images is used to generate the activation levels for the phase simulations.

Cortical measurements show that the distribution of non-directional monophasic simple cells is roughly uniformly distributed between zero-, first- and second order Gaussian derivatives (Young and Lesperance, 2001). We performed the simulations presented here also with mixed receptive fields of zero-, first- and second-order Gaussian derivatives and obtained similar results. We present here only results with second order Gaussian derivatives, because this reduces the number of model parameters drastically.

#### 2.4.2. Autoencoder filters

As a comparison to these regular hand-designed Gabor filters we analyze the oscillatory network based on activation levels generated by unsupervised learned autoencoder weights. A good overview of the concepts described in this section can be found in Le et al. (2011b), where the authors analyze different optimization methods for convolutional and sparse autoencoders. An autoencoder learns a higher level representation from the stimulus statistics such that the input stimuli can be reconstructed from the hidden representations. In addition, we optimize the sparsity of the activation levels in this representation, which was shown to learn connection weights which resemble receptive fields in the visual cortex (Olshausen and Field, 1996; Hinton, 2010; Le et al., 2011a).

**Figure 2:**
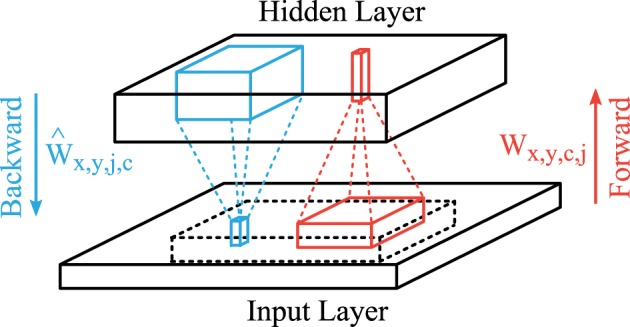
**Structure of the convolutional autoencoder**. Convolutional forward weights (red) compute the hidden layer activation levels and convolutional backward weights (blue) generate the reconstruction of the given input. The reconstruction layer is compared to the centered part (dashed block) of the input layer.

A common trick in unsupervised learning in neural networks are shared connection weights to reduce the number of parameters that have to be learned, which can be accomplished by a convolutional feed-forward network in the case of images (LeCun et al., 1998; Lee et al., 2009). The structure of our convolutional autoencoder is shown in Figure 2. The feedforward projections that generate the activation of feature map *j* consist of convolutional filters *W*_*x,y,c,j*_ (red lines in Figure 2) with input features *c* ∈ {1,2,3} (rgb-colors) and a bias term *b*_*j*_ and is followed by a sigmoidal activation function. Therefore, the hidden layer activation map of feature *j* ∈ {1, 2, …, *J*} is described by

(7) $$h_{x,y,j}=f\left(\sum_{c=1}^{3}W_{x,y,c,j}*v_{x,y,c}+b_{j}\right).$$

The hidden layer activation *h* of each input image sample is also a 3 dimensional block (horizontal and vertical image dimensions and the feature type). The weight matrix *W* is a 4 dimensional structure which describes the connection weights from a convolutional input block to one output column in the hidden layer. The convolutional image operations (*) are applied in the image directions *x* and *y* between all combinations of input feature maps *c* and all output feature maps *j*.

We use linear activation functions for the backward projections (blue lines in Figure 2) so that the output matches the scale of the input images (zero-mean). We use another set of weights *Ŵ*_*x,y,j,c*_ and bias terms ${\hat{b}}_{c}$ to describe these backward connections. Therefore, the activation in the reconstruction layer is given by

(8) $${\hat{v}}_{x,y,c}=\sum_{j=1}^{J}{\hat{W}}_{x,y,j,c}*h_{x,y,j}+{\hat{b}}_{c},$$

where *J* = 100 is the number of different feature types. During the learning stage only the valid part (no zero padding) of the convolutions are used for the forward and backward projections to avoid edge effects of the image borders on the learned weights. Similar to the Gabor filters the convolutional filters have a size of 12x12 pixels and are applied using a stride of 2 pixels leading to a reduction in the resolution of the hidden layer.

We use the sum of 3 optimization functions to learn the forward and backward weights of the autoencoder. The first optimization term which is minimized is the reconstruction error averaged over all positions and training samples *s* and is given by

(9) $$\Psi_{1}={\langle \frac{1}{2}{\left\|{\hat{v}}_{x,y,c}^{\left(s\right)}-v_{x,y,c}^{\left(s\right)}\right\|}^{2}\rangle }_{x,y,s}.$$

The second term optimizes the sparseness of the hidden units as described by Hinton (2010) and Le et al. (2011a) with

(10) $$\Psi_{2}=\beta \cdot \sum_{j}\text{KL}\left(\tilde{h}\|{\langle h_{x,y,j}^{\left(s\right)}\rangle }_{x,y,s}\right),$$

where *KL* is the Kullback-Leibler-divergence between two Bernoulli distributions with expected values $\tilde{h}$ and 〈*h*^(*s*)^_*x,y,j*_〉_*x,y,s*_. We set the desired average activation $\tilde{h}$ = 0.035.

The third term is a weight decay (L2-norm) of all forward and backward weights and is given by

(11) $$\Psi_{3}=\frac{\lambda }{2}\cdot \left(\sum_{x,y,c,j}W_{x,y,c,j}^{2}+\sum_{x,y,j,c}{\hat{W}}_{x,y,j,c}^{2}\right).$$

This optimization term pushes all connection weights toward zero such that only the connections which help to extract useful features remain. Therefore, it provides a regularization mechanism during learning.

For the simulations presented in this paper we use a relative weighting between these optimization functions given by β = 90 and λ = 0.3. The gradients of the optimization functions are calculated using back propagation of error signals and were checked using numerical derivatives. The sum of the three terms described above is minimized with the limited memory Broyden-Fletcher-Goldfarb-Shanno algorithm (L-BFGS), which uses an approximation to the inverse Hessian matrix (Liu and Nocedal, 1989). We use the *minFunc* library of Mark Schmidt^1^ with default parameters for line search with a strong Wolfe condition. We use L-BFGS because it converges much faster in comparison to standard gradient descent, especially in the case of autoencoders with sparseness constrains (Le et al., 2011b). Another advantage of L-BFGS is that extensive tuning of learning parameters as in standard gradient descent methods is not necessary.

The training data consists of 1000 color patches (60 × 60 pixels) sampled from the folder *05june05_static_street_boston* of the LabelMe database (Russell et al., 2008). This corresponds to 625.000 training samples per convolutional fragment where the forward weight matrix is applied. After 500 iterations the features are mostly oriented patches and sensitive to different colors.

#### 2.4.3. Regularization of activation levels

Although the Gabor and autoencoder filters are both followed by a sigmoidal activation function, we further sparsify the activation levels *h*_*x,y,k*_ with feature types *k* ∈ {1..*K*} in a similar way to local cortical circuitry. We want to constrain the number of active neurons, rather than the mean activation levels. Therefore, we subtract at each image position the average local activation levels. Subsequently a half-wave rectification is applied to constrain the activation levels again to the positive domain with roughly half of the neurons inactivated:

(12) $${\tilde{h}}_{x,y,k}=\text{max}\left(0,h_{x,y,k}-\sum_{j=1}^{K}h_{x,y,j}\right).$$

Consequently the hard sparseness (Rehn and Sommer, 2007) is artificially increased and these inactivated neurons do not take part in the coupling of phase oscillations (see section 3.1). Thereby the number of possible interactions in the phase simulations is reduced.

As a last step we have to normalize the activation levels at every image position similar to local contrast adaptation in the visual system. We want to make sure that the overall local activation is uniform over the visual field such that an efficient coding of regions of high contrast and regions of low contrast is possible simultaneously. Therefore, we divide all activation levels by the sum of activations over all features at each image location:

(13) $$g_{x,y,k}=\frac{{\tilde{h}}_{x,y,k}}{\sum_{j=1}^{K}{\tilde{h}}_{x,y,j}}.$$

As a result we have sparse activation maps with a large proportion of inactive neurons and the same average local activations at all image positions.

## 3. Results

In a first step we analyse the properties of the activation patterns induced by the natural images (section 3.1). Subsequently we evaluate the correlation statistics of these induced feature activations (section 3.2) and the resulting sparse connectivity pattern (section 3.3). Based on this connectivity pattern we show simulations of the coupled phase oscillator model and the resulting dynamic phase maps (section 3.4). Finally, evaluations of these binding maps are presented based on human labeled segmentation masks (section 3.5).

### 3.1. Sparseness of activation

The simulation of the coupled phase oscillators is based on the activation levels that were generated from natural images. The phase coupling is highly dependent on the type of feature representation that is used to generate the activation levels. The first reason is that the connectivity is based on the correlation between features. The second reason is that also the actual strength of the dynamic coupling is proportional to the current activation levels. Therefore, the statistics of activation plays a crucial role in the formation of the dynamic binding maps.

**Figure 3:**
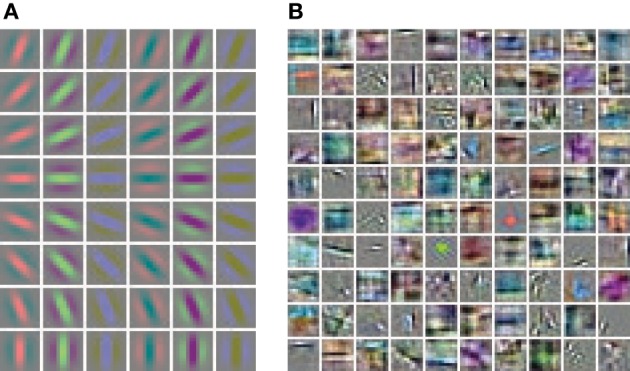
**Receptive fields of the feed-forward connections generating the activation levels for the phase simulations. (A)** The regular Gabor filters are generated with 8 different orientations and 6 different color channels. **(B)** The convolutional autoencoder weights are learned by optimizing the reconstruction cost, sparseness and weight decay.

Hand labeled photographs of suburban scenes from the LabelMe database (Russell et al., 2008) are used to generate feature representations with the linear convolutional forward weights followed by a sigmoidal function. The linear convolutional kernels of the Gabor receptive fields contain only one spatial frequency and equally spaced orientations (Figure 3A). In contrast, the learned weights of the sparse autoencoder (Figure 3B) cover a diverse set of spatial frequencies, colors and orientations.

**Figure 4:**
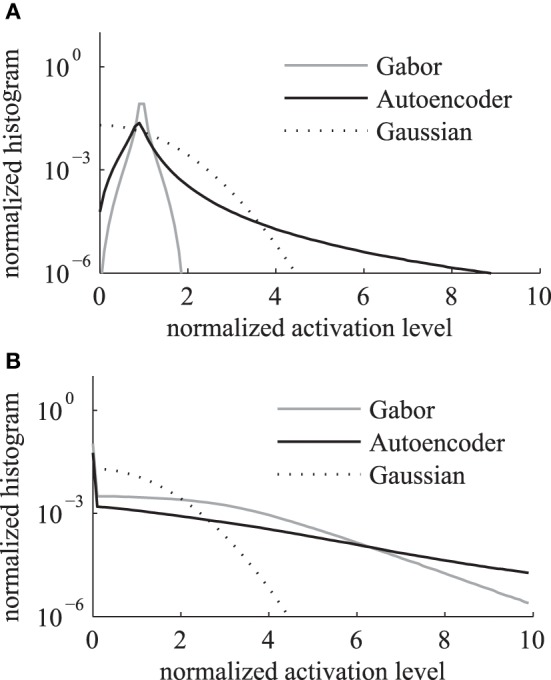
**Histogram of activation levels averaged over all feature types**. The distributions of activation levels are compared to a Gabor distribution. **(A)** After sigmoidal activation function. **(B)** After mean subtraction, half-wave rectification and division by the sum.

We compare the activation levels of features obtained with the regular Gabor weights and the autoencoder weights. A very important characteristic of neuronal activations is the level of sparseness. A high level of activation sparseness means that the neuron is most of the time very silent and only rarely very active. This analysis of sparse coding should not be confused with the graph theoretic sparseness which will be analyzed in section 3.3. A qualitative comparison of the activation histograms (Figure 4A) shows that the autoencoder activations are sparser compared to the Gabor activations. The phase model is based on the assumption that the activation is restricted to the positive domain. Note that this is in contrast to many normative models of early visual processing which assume a feature code with a Gaussian distribution with zero mean. Furthermore, in our model we are mostly interested in the “hard sparseness” of the activation levels, meaning that the activation is most of the time exactly zero and only rarely very high (Rehn and Sommer, 2007). A comparison with a Gaussian distribution restricted to the positive domain with the same mean (dashed line in Figure 4A) reveals that the feature activations after the sigmoidal activation function are not necessarily sparse in the context of a positive distribution with this hard sparseness criteria.

The sigmoidal activation function is followed by the subtraction of mean, rectification and the division by the sum over all features. The resulting histograms of these activation levels (Figure 4B) show an increased hard sparseness for both types of receptive fields. These additional preprocessing steps are similar to local regulatory mechanisms in the cortex.

A quantitative evaluation of the sparseness of the activation levels is given by the kurtosis. We use the standard measure of excess kurtosis but without mean normalization because the phase model assumes a non-negative feature coding by activation. Therefore, we evaluate the hard sparseness of feature type *j* with activation levels *h*^(*s*)^_*x,y,j*_ by the kurtosis of a zero-centered distribution given by

(14) $${\text{kurt}}_{j}=\frac{{\langle {\left(h_{x,y,j}^{\left(s\right)}\right)}^{4}\rangle }_{x,y,s}}{{\left({\langle {\left(h_{x,y,j}^{\left(s\right)}\right)}^{2}\rangle }_{x,y,s}\right)}^{2}}-3,$$

where 〈.〉 is the mean over all image positions (*x*, *y*) and image samples *s* from the labelMe database. The estimated median kurtosis over all receptive field types increases for the activations *g* after the normalization steps described above in comparison to the activations *h* before the normalizations (Table 1). A comparison with a Gaussian distribution, which has a kurtosis of 0, reveals that the additional activation functions indeed increase the sparseness and lead to a leptokurtic distribution of activations. Overall the activations generated by the autoencoder are more sparse in comparison with the hand designed Gabor filters.

**Table 1:** **Median kurtosis of feature activations**.

	**After sigmoid activations *h_x,y,k_***	**After normalizations^1^*g_x,y,k_***
Gabor	−1.96	2.61
Autoencoder	−0.63	11.62

1 *After the subtraction of mean, half-wave rectification and division by the local sum of the new activation levels*.

The additional activation functions are crucial for the subsequent phase simulations. The mean subtraction and half-wave rectification increase the hard sparseness of activations. This reduction in the number of active neurons leads to a reduction in the number of active tangential phase connections. Therefore, the features in the input image do not only multiplicatively modulate the strength of the phase interaction but also deactivate many phase connections entirely leading to a completely new effective tangential connectivity pattern.

### 3.2. Statistics of horizontal cross-correlations

**Figure 5:**
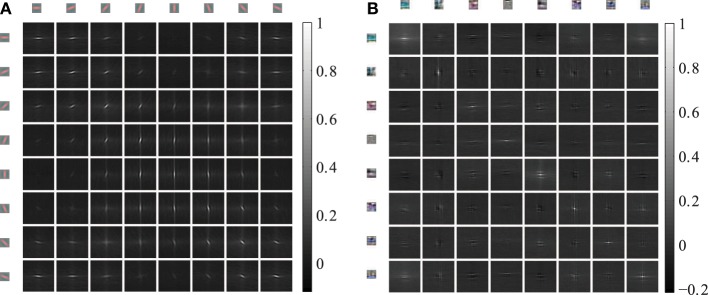
**Cross-correlations between different feature activations shifted in visual space**. The shown cross-correlations are based on the activation levels induced by natural images. Only a subset of 8 features is shown. The patches on the top and left row show the forward weight matrix of the receptive fields. The other patches show the spatial correlation between these features. The feature weights are shown at the same spatial scale as the shifts in the cross-correlations. **(A)** The correlations between 8 oriented Gabor filters of one of the 6 color channels are shown. **(B)** The correlations between 8 randomly choosen autoencoder features are shown.

The horizontal connections between the coupled phase oscillators are sampled from the cross-correlations of induced activation levels as described in equation 5. Therefore, we describe the horizontal correlations in this section and evaluate the anisotropy of receptive field types. The 4 dimensional cross-correlation tensors ρ^(*k,m*)^_*x,y*_ as defined in section 2.3 are shown in Figure 5 for 8 feature types. The Gabor receptive fields have a more regular correlation matrix (Figure 5A) compared to the learned autoencoder receptive fields (Figure 5B). The correlations between the activations of Gabor receptive fields are itself similar to high frequency Gabor functions. In contrast, the receptive fields learned by the autoencoder capture different spatial frequencies and a variety of different colors which is also reflected in the spatial cross-correlations. In both cases the horizontal cross-correlations extend over visual space up to three times the receptive field size. This suggests that the correlations indeed comprise higher-order correlation statistics of the natural images and not only interactions between overlapping receptive fields.

**Figure 6:**
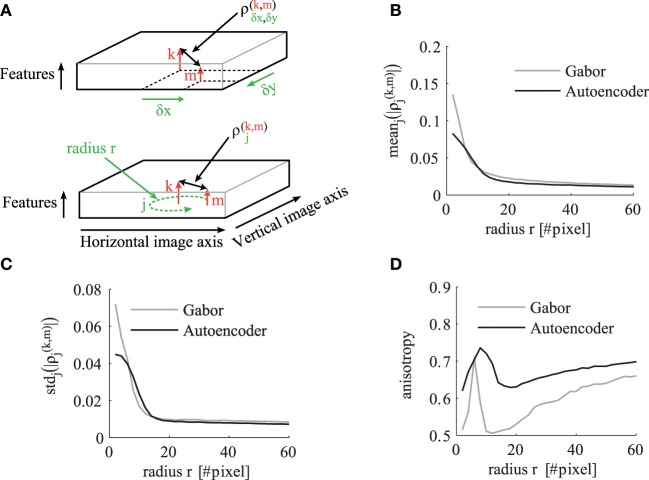
**The statistics of the correlation matrix evaluated for different distances *r* in visual space. (A)** Schematic to illustrate the indices of the correlation tensor. In the top schematic the correlation tensor is indexed by horizontal (x) and vertical (y) offsets in visual space. In the bottom schematic the correlation tensor is indexed by *j* ∈ *R_r_* for a certain distance *r* in visual space. The other panels compare the correlation tensor of Gabor filters (gray) and autoencoder filters (black) for different distances *r*. All shown statistics are averaged over all pairs of receptive field types *k* and *m*. **(B)** Mean over all directions. **(C)** Standard deviation over different directions for a certain pair of feature types. **(D)** The anisotropy averaged over all pairs of receptive fields as described in the main text.

To analyze and compare the correlation tensor of the autoencoder and the Gabor filters, we calculate statistics for different correlation distances in visual space. The indices of the tensor are illustrated in the schematic in Figure 6A. For each distance *r* in visual space we calculate statistics over ρ^(*k,m*)^_*j*_ where

(15) $$j\in R_{r}:=\left\{\left(x,y\right)\in {\mathbb{Z}}^{2}|r-\frac{1}{2}\leq \sqrt{x^{2}+y^{2}}<r+\frac{1}{2}\right\}.$$

The mean absolute value of the cross-correlations decreases for larger correlation distances *r* as shown in Figure 6B. The mean standard deviation of these absolute correlation values over different spatial directions also decreases but with a steeper slope (Figure 6C). To make a relative statement about the isotropy in the correlation tensor we also calculate the coefficient of variation over different directions. Therefore, we define the average anisotropy at radius *r* as

(16) $$\text{anisotropy}(r):={\langle \frac{{\text{std}}_{j\;\in \;R_{r}}\left(\rho_{j}^{\left(k,m\right)}\right)}{{\text{mean}}_{j\;\in \;R_{r}}\left(\rho_{j}^{\left(k,m\right)}\right)}\rangle }_{k,m}$$

This mean anisotropy averaged over all pairs of receptive field types has a local maximum at visual distances of around 8–10 pixels (Figure 6D). This suggests that the short range phase connections over this distance help more in the synchronization of fine structures. The anisotropy has a local minimum at distances around 15–16 pixels, where more long range phase connections are dominantly used to fill-in segment pixels with similar colors.

### 3.3. Sparsely connected oscillator network

The correlation values are used to sample the sparse connections for the simulations of coupled phase oscillators. We restrict the sampled connectivity pattern in simulations of natural scenes to 200 synchronizing and 200 desynchronizing afferent connections per neuron if not stated otherwise. The phase simulations of natural image scenes are run in a network of 200×150×48 neurons for Gabor features or 200×150×100 for autoencoder features respectively. Therefore, the percentage of connections that are actually formed compared to all possible connections assuming full connectivity is approximately 0.014% in the case of Gabor features and 0.007% for autoencoder features. Thus, this procedure leads to a very sparse connectivity in comparison to a network of all-to-all interactions.

We evaluate the sampled connectivity based on natural image statistics using graph theoretic measures. The connectivity structure is represented as a graph *G* = (*H, E*) as described in section 2.3. We compute the statistics not only over the graph of all connections *E* but also for the subgraph of synchronizing connections $E^{+}:=\left\{e_{x,y}^{\left(j,k\right)}\in E|e_{x,y}^{\left(j,k\right)}=+1\right\}$ and the subgraph of desynchronizing connections $E^{-}:=\left\{e_{x,y}^{\left(j,k\right)}\in E|e_{x,y}^{\left(j,k\right)}=-1\right\}$ individually.

For a graph with edges *E* we calculate the fraction of intra-feature connections as

(17) $$\mu =\frac{\left|\left\{e_{\delta x,\delta y}^{k,m}\in E|k=m\right\}\right|}{\left|\left\{e_{\delta x,\delta y}^{k,m}\in E|k\neq m\right\}\right|}\cdot 100\%.$$

The most obvious observation is that the fraction of intra-feature connections is larger for synchronizing connections in comparison to the desynchronizing connections (Table 2). The reason is that positive correlations, which are used to sample these synchronizing connections, are stronger between the same feature type shifted over visual space. In contrast negative correlations and thus desynchronizing connections are less likely to occur between the same feature type shifted over visual space. Another observation is that the fraction of intra-feature connections of the Gabor features is roughly twice as large as in the case of the autoencoder features. The reason is that we use 100 autoencoder features and only 48 Gabor features while the total number of sampled synchronizing and desynchronizing connections per feature remains constant.

**Table 2:** **Graph theoretic statistics of the sparse connectivity pattern**.

	**Gabor**			**Autoencoder**		
	**All *E***	**Sync. *E*^+^**	**Desync. *E*^−^**	**All *E***	**Sync. *E*^+^**	**Desync. *E*^−^**
Fraction of intra-feature connections μ	6.49%	12.87%	0.10%	3.12%	6.17%	0.07%
Global clustering coefficient γ (in 10^−3^)	3.21	3.53	0.64	3.18	2.49	1.27
Global clustering coefficient random γ_random_ (in 10^−3^)	2.35	1.11	1.25	2.18	1.03	1.18
Mean shortest path length λ	2.01	2.60	2.46	2.17	2.63	2.59
Mean shortest path length random λ_random_	2.02	2.53	2.47	2.15	2.62	2.56
Small world index σ_sw_	1.37	3.09	0.51	1.44	2.39	1.07

A more elaborate evaluation of the sampled connectivity of our network can be done using the clustering coefficient and the small-world characteristics (Watts and Strogatz, 1998; Humphries et al., 2006), which are also shown in Table 2. To define the local clustering coefficient in an infinite graph *G* = (*H, E*), we analyze the connectivity of the neurons in a generic feature column at position (*x*, *y*) = (0,0). We define the neighbors of neuron *g*_0,0,*k*_ coding feature type *k* ∈ {1..*K*} as the set of all neurons which are directly connected in the graph as

(18) $$N_{k}=\left\{g_{x,y,m}\in H|e_{x,y}^{k,m}\in E\vee e_{-x,-y}^{m,k}\in E\right\},$$

where we consider outbound (*e*^*k,m*^_*x,y*_) and inbound (*e*^*m,k*^_−*x*,−*y*_) connections of the neuron. Then we define the local clustering coefficient of a feature type *k* in our network as the fraction of the number of direct connections between neighbors to the number of pairs of neighbors:

(19) $$\gamma_{k}=\frac{\left|\left\{e_{x,y}^{m,n}\in E|g_{\tilde{x}+x,\tilde{y}+x,m}\in N_{k}\wedge g_{\tilde{x},\tilde{y},n}\in N_{k}\right\}\right|}{\left|N_{k}\right|\;\cdot \;(\left|N_{k}\right|-1)}$$

We show the global clustering coefficients γ = < γ_*k*_ >_*k*_ for our sampled networks comprising only the synchronizing, only the desynchronizing or all connections in the second row of Table 2.

The evaluation of the graph comprising all connections shows that the mean clustering coefficient is roughly the same for the Gabor and the autoencoder features. But the evaluation of graphs individually reveals that the clustering coefficient of only the synchronizing graph is higher for the Gabor features in comparison to the autoencoder features. And reciprocally, the desynchronizing connections show a stronger clustering in the case of autoencoder features. An explanation for this difference is that the autoencoder learns a more diverse set of receptive fields by optimizing the reconstruction error. In comparison, the regular Gabor receptive fields cover only predefined colors, spatial frequencies and orientations, which are not optimized to cover a broad range of statistics in the input images. Therefore, the correlation structure in the Gabor activations shows stronger clustering. For comparison, we also show the corresponding clustering coefficients γ_random_ of the equivalent networks with the same connection lengths (measured in pixel distance) but rotated by random angles and connected to random features.

We can further use the small-world index to measure the capability of neurons in our network to reach other neurons via a small number of interaction steps. The small-world index is a quantitative definition of the presence of abundant clustering of connections combined with short average distances between neuronal elements, proposed by Humphries et al. (2006). It can characterize a large number of not fully connected network topologies. The connectivity within the 3-dimensional grid of our model is sampled such that it is invariant to shifts in the two image dimensions. Therefore, we have to slightly adapt the small-world index for our infinite horizontal sheet consisting of feature columns with identical connection patterns. We use the definition of the small-world index

(20) $$\sigma_{\text{sw}}=\frac{\gamma /\gamma_{\text{random}}}{\lambda /\lambda_{\text{random}}},$$

where the shortest path lengths λ and λ_random_ measure the number of network hops needed to connect two neurons within our sampled network and a random network respectively. We use the average over all shortest path lengths between all pairs of neurons within one feature column. A network graph must have a small-world index σ_*sw*_ larger than one to meet the small-world criteria. The evaluations show that the graph comprising the synchronizing connections exhibits small-world properties while the desynchronizing connections are closer to a random connectivity and do not exhibit small-world properties (Table 2). The small-world property might be helpful in the synchronization of distant neurons.

### 3.4. Phase simulations

The resulting connectivity pattern is used in the phase simulations. All shown simulations of the coupled phase oscillator networks are initialized with random phase variables. The activation levels are only set once in the beginning and remain the same throughout the phase simulations. During the simulations attractors are formed in the phase space and are localized in certain image regions.

**Figure 7:**
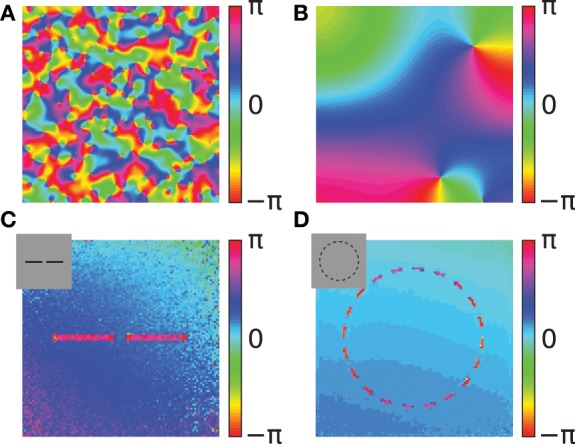
**Simulations of the coupled phase oscillator model**. The phase variables are shown as color hue. **(A,B)** Phase simulations of a 2-dimensional grid of 200x200 neurons with uniform activation levels after 100 network iterations. A regular local connectivity is used with maximum length 3 **(A)** and 10 **(B)**. Simulations in **(C,D)** use the Gabor receptive fields and horizontal oscillator connectivity from the correlation statistics. The input images are artificial stimuli of two collinear aligned bars **(C)** and of a dashed circle **(D)** and are shown as an inset in the upper left corner. The shown phase maps in **(C,D)** are the circular mean of the phase variables weighted by the activation of the corresponding features. Panel **(C)** shows the average phase after 20 network iterations and panel **(D)** after 40 network iterations.

A simulation of the coupled phase oscillator model with localized connectivity and with uniform activation levels shows that pinwheel structures will form in the phase map (Figures 7A,B). The connectivity length in the network determines the scale of the pinwheels. During the simulation these pinwheels attract each other and annihilate (Wolf and Geisel, 1998). The probability of the formation of pinwheels decreases for network connectivity patterns that are less locally dense but more sparse and spread out.

In the next simulations we use several feature types to encode different aspects of the input images. To visualize the resulting 3-dimensional structure of phase variables φ_*x,y,k*_ we calculate the circular mean at each image position weighted by the corresponding activation levels:

(21) $$\varphi_{x,y}^{\text{avg}}:=\text{arg}\left(\sum_{k}g_{x,y,k}e^{i\varphi_{x,y,k}}\right),$$

where arg is the complex argument. We show the average phase variables φ^avg^_*x,y*_ coded as color hue to visually represent the circular structure of the phase.

We use two simple artificial stimuli to demonstrate the basic function of the phase simulation in the presence of structure in the activation variables (Figures 7C,D). The stimuli of these simulations are artificially generated grayscale images containing bar segments and circle segments (insets in Figures 7C,D). The connectivity in both simulations is based on Gabor receptive fields with horizontal connectivity obtained from statistics of natural images. In the simulation of two collinear aligned bars the phase of the neurons coding the two bars are synchronizing although the two bars are not directly connected in the image (Figure 7C). This suggests that the simulation can implement Gestalt laws of grouping, because neurons are grouped together by having the same phase value. Specifically, a human observer could interpret these two bars as one single continues line. Therefore, the simulation can be interpreted as implementing the Gestalt law of continuity because the neurons that are coding the two bars have the same phase. Please note, that in the simulation the gap between the two bars is not filled in because our model does not incorporate any feedback from the phase variables to the activation variables. In this study we focus on relational coding by phase variables and therefore neglect any recurrent dynamics in activation variables.

The other simulation uses a dashed black circle as input (Figure 7D). The phase map shows that all segments of the circle are synchronizing to the same phase value. The synchronized state of the circle means that the phase variables at different segments of the circle code the global attribute and bind the individual circle segments together. Similarly, humans usually perceive the circle segments all together as one single object. This indicates that the phase simulation can also implement the Gestalt law of closure. Depending on the initialization of random phase variables, cases exist where the circle does not synchronize to one coherent phase but forms a continuous phase progression one or multiple times from 0 to 2π. On one hand these simulations reproduce the previous studies demonstrating binding properties of coupled neural oscillators. On the other hand, in these simulations the connectivity is learned based on natural stimuli and not hand crafted. Hence, it demonstrates that these Gestalt properties are learned from the statistics of natural stimuli.

**Figure 8:**
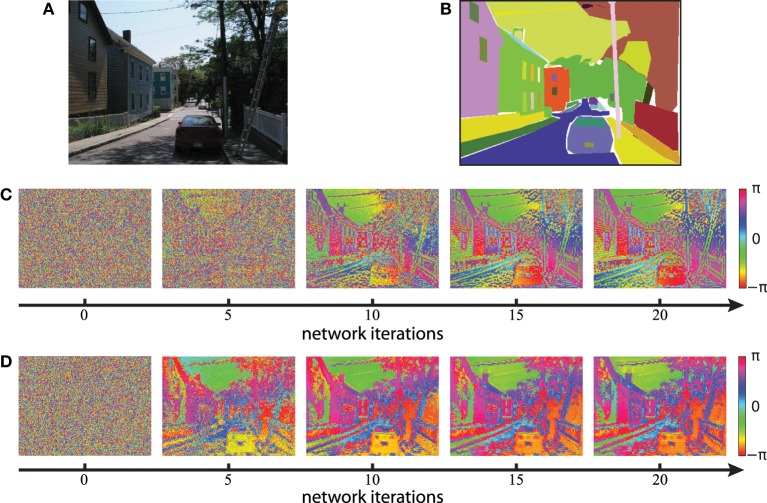
**Phase simulations of a natural image of a suburban scene. (A)** A natural image from the LabelMe database is used as the input to generate neuronal activation maps. **(B)** The LabelMe images are accompanied by overlapping segmentation masks of labeled image regions. **(C,D)** The circular mean of the phase maps evaluated at different network iterations. Gabor filters were used in **(C)** and autoencoder filters in **(D)**.

We next evaluate the concept of binding by synchrony also on natural visual scenes. All following simulations in this paper use color images from the LabelMe database (Russell et al., 2008) and either the Gabor filters or the autoencoder filters to generate the activation levels for the network. An example of a suburban scene is shown in Figure 8A with the corresponding human labeled segmentation masks in Figure 8B. We use the time constant τ = 1/3 for the simulations based on Gabor filters and τ = 1/30 for the simulations based on autoencoder filters. These values were chosen such that per iteration of the classical Runge-Kutta solver the phase of not more than 1% of all neurons changes more than π/2. The units of these time constants are arbitrary because our model of coupled phase oscillators describes the change in phase independent of the oscillation period. Examples of the resulting phase maps are shown in Figure 8C for Gabor activations and Figure 8D for autoencoder activations. The phase maps of simulations using autoencoder weights are blurred compared to the Gaborfilters because the peak of the receptive fields are not necessarily centered within the convolutional weight matrix, leading to shifts in visual space between different feature maps at segment boundaries. Yet in both examples an intuitive segmentation of the original can be recognized again in the distribution of phase values. We see a constantly increasing phase synchrony in labeled segments. This example suggests that high-level image objects are likely to synchronize to a coherent phase.

### 3.5. Evaluation of phase maps

We evaluate the simulated dynamic phase maps and compare them with human labeled binary segmentation masks of high level image objects from the LabelMe database. We begin with an evaluation of the resulting phase maps independently from the labeled image masks to show global properties of the coupled phase oscillator model and the influence of the number of horizontal connections (section 3.5.1). This is followed by an evaluation of the phase synchrony within labeled segments with respect to the surrounding of the segments (section 3.5.2). Finally a local evaluation of the phase maps at the boundaries of labeled segments is presented (section 3.5.3).

#### 3.5.1. Phase synchrony

Segmentation and binding of neurons in the network can only be achieved if the phase variables are not random but also not completely synchronized. Therefore, we will first evaluate the local phase synchrony independent of segments in the image. We define the synchrony in a population *M* of neurons as

(22) $$p_{M}=\left|\frac{\sum_{m\in M}g_{m}\cdot e^{i\varphi_{m}}}{\sum_{m\in M}g_{m}}\right|,$$

where *M* is defined as a set of 3-dimensional indices describing the position of the neurons.

**Figure 9:**
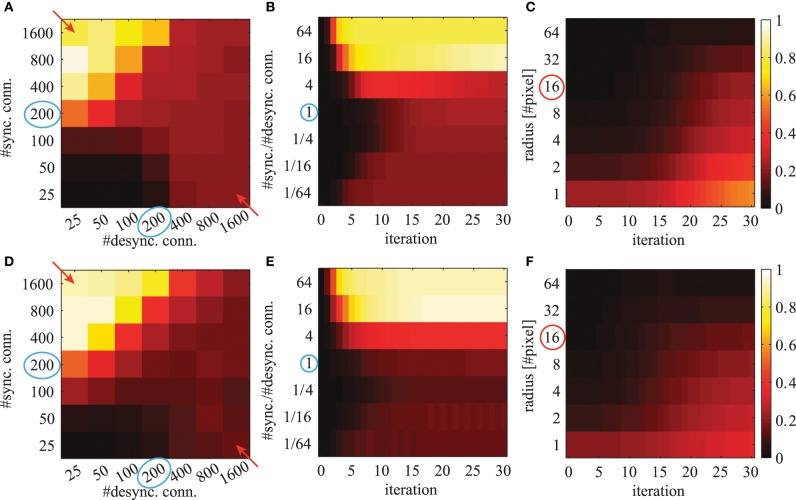
**The averaged local phase synchrony in circular image regions for different simulation parameters**. All evaluations are based on the activation levels obtained from the image shown in Figure 8A. Simulations in the top row **(A–C)** are based on Gabor weights; simulations in the bottom row **(D–F)** are based on autoencoder weights. Blue circles indicate the standard parameters for subsequent evaluations. Colorbars of all panels are the same and shown on the right. The panels in the left column **(A,D)** show the phase synchrony after 20 iterations for different number of excitatory and inhibitory phase connections per neuron. The panels in the center column **(B,E)** show the phase synchrony for different ratios of excitatory to inhibitory connections as a function of network iterations. These ratios correspond to the diagonal elements marked with red arrows in panels **(A,D)**. And the shown time course of the average phase synchrony values are from the same simulations. In the right panels **(C,F)** the phase synchrony is shown for different sizes of the local circular region of the evaluations. The red circle indicates the radius which was used in the evaluations shown in the other panels.

In this section we analyze the simulation shown in Figure 8 in more detail and evaluate how the number of synchronizing and desynchronizing connections effects the phase synchrony. We evaluate the local phase synchrony at image position (*x*, *y*) for a certain radius *r* by calculating *pM_x,y,r_* for neurons at positions

(23) $$\begin{array}{l} M_{x,y,r}=\{(\tilde{x},\tilde{y},k)|{\left(x-\tilde{x}\right)}^{2}+{\left(y-\tilde{y}\right)}^{2}<r^{2},\\ \;\left(x,y)\in {\mathbb{N}}^{2},k\in \{1..K\right\}\}, \end{array}$$

where *K* is the number of feature maps. We average this quantity over all possible image positions (*x*, *y*). This mean local phase synchrony is shown in Figure 9 for simulations using different number of connections, different iterations and for different radii *r*.

When the network has reached a steady state, the mean local phase synchrony depends on the number of synchronizing and desynchronizing connections (Figures 9A,D). The number of synchronizing connections increases the average local phase synchrony. In contrast, the number of desynchronizing connections can increase or decrease the average local phase synchrony depending on the number of synchronizing connections. At first sight, this may be counterintuitive. In the case of few synchronizing connections, the desynchronizing connections repel the associated phase variables from each other. This ultimately leads to a clustering in the circular phase space evoked by desynchronizing interactions. In the case of more synchronizing connections, the main force driving the network are attractor states and therefore desynchronizing connections decrease the overall phase synchrony.

The phase synchrony in the steady state condition increases with the ratio between synchronizing and desynchronizing connections up to a ratio of 16 times more synchronizing than desynchronizing connections (Figures 9B,E). Interestingly, the phase synchrony in the steady state condition decreases again in simulations with more than 800 synchronizing connections and very few desynchronizing connections. During the transient phase a very low or high ratio leads to a faster convergence to a more synchronized state. The slowest convergence is achieved at the cases with 4 times more desynchronizing connections or when the number of synchronizing and desynchronizing connections is balanced.

The phase simulations show synchronization behavior at a large variety of different spatial scales (Figures 9C,F). The level of synchrony at the steady state decreases for increasing radius of the phase synchrony evaluation. At all spatial scales the time to reach the steady state synchrony level is roughly the same. Only very localized regions over 1-2 pixel distances show a slightly faster convergence to the final phase synchrony level. When not otherwise stated we select in all simulations and evaluations an intermediate parameter range with balanced synchronizing and desynchronizing connections leading to rich dynamics. These standard parameters are marked with blue circles in Figure 9.

#### 3.5.2. Segmentation index

The dynamic binding and segmentation of the simulated phase maps of natural images are evaluated using hand labeled segmentation masks. Here a baseline is necessary to accommodate for the higher probability of synchronization between neurons that are close by. Consequently we use the labeled image masks on the corresponding simulated phase maps and compare them to a baseline using the same image masks on simulations of different non-matching images.

**Figure 10:**
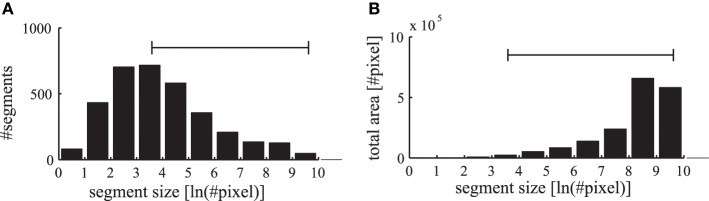
**Statistics of labeled image segments. (A)** The histogram of evaluated segments from the LabelMe database for different segment sizes is shown. **(B)** The total area occupied by the segments in the corresponding bins. The range of segment sizes (36–15000 pixels) that are used for subsequent evaluations are marked with a horizontal bar.

The segmentation masks in the LabelMe database are specified as polygons on the images that are initially reduced in our simulation to a resolution of 400×300 pixels. The convolutional forward projections lead to a further reduction in the feature representation to a grid of 200×150 pixels. Therefore, we restrict the evaluations of the phase maps to segmentation masks which contain at least as many pixels as the specified patch size of the forward projections (6×6 neurons corresponding to 12×12 pixels in the input image). In addition, segments occupying more than half of the respective images are excluded to allow evaluations against a baseline synchrony of the surrounding regions. The range of labeled segments which is used in our evaluations is shown as a horizontal bar in Figure 10. Only in evaluations where the segment sizes are explicitly stated, we also evaluate these otherwise excluded very small and very large segments.

The number of labeled segments in the database decreases for larger segment sizes (Figure 10A). Yet the total area occupied by segments in the different bins increases for larger segment sizes (Figure 10B). Therefore, when applying labeled masks to non-matching images small segments are highly likely to fall into large segments where a large number of tangential connections is functionally active. Consequently the phase synchrony within labeled segments is not a sufficient baseline for an unbiased comparison with simulations of non-matching images. Therefore, we need a baseline to control for the unequal distribution of segment sizes and their occupied region in the images.

**Figure 11:**
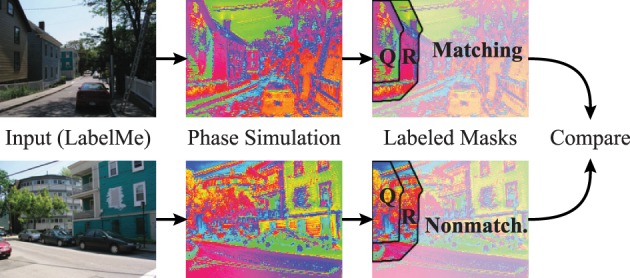
**Evaluation using hand labeled image masks**. The evaluations compare the segmentation index of matching simulations and segmentation masks **(top row)** to a baseline of non-matching simulations and segmentation masks **(bottom row)**. The images from the LabelMe database (left column) are processed using the forward projections. The resulting features are used to simulate the phase of the coupled neural oscillators **(middle column)**. The segmentation index of these phase maps are then evaluated using the segmentations masks from the LabelMe database **(right column)**. The evaluation of the house in the top left is here shown as an example. The segmentation index compares the phase synchrony in the hand labeled region of the house (*Q*) to a baseline phase synchrony within the neighborhood (*Q* ∪ *R*).

To accommodate for the statistics of segment sizes in the evaluation of the matching and non-matching natural scenes, we define a segmentation index (Figure 11) that sets the phase synchrony in segments into the context of the surrounding neurons. Concretely, the segmentation index evaluates how the phase of neurons inside of segments is more or less synchronized compared to the synchrony of random neurons inside and outside of the segment. The neighborhood *N* of a segment *Q* is generated using a diamond shaped grow operation on the segmentation mask repeatedly until the number of neurons in *N* is doubled compared to the original segment *Q*. Therefore, *N* is the union of the segment *Q* and the surrounding *R* of the segment (*Q* and *R* are annotated in the example shown in Figure 11).

We calculate the phase synchrony values *p*_*Q_j_*_ and *p*_*N_l_*_ for random subsets *Q_j_* ⊂ *Q* and *N_l_* ⊂ *N* where *j*, *l* ∈ {1, …, 100} and *Q_j_*, *N_l_* ∈ ℕ^1000^. We define the segmentation index of segment *Q* as the difference between the mean synchrony within the segment *Q* to the mean synchrony in the neighborhood *N* = *R* ∪ *Q*:

(24) $$\kappa (Q,N)={\langle p_{Q_{j}}\rangle }_{j}-{\langle p_{N_{l}}\rangle }_{l}.$$

**Figure 12:**
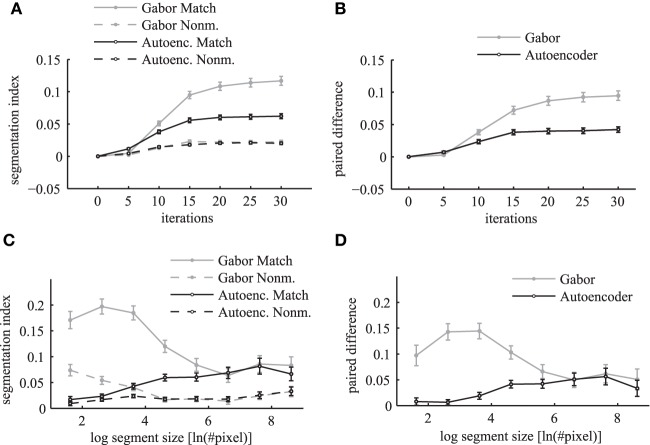
**Segmentation index**. The mean segmentation index is shown as a function of network iterations averaged over all segments with more than 36 pixels in the top panels **(A,B)**. The segmentation index is shown as a function of different segment sizes after 20 network iterations in the bottom panels **(C,D)**. The panels on the left side **(A,C)** show the evaluations for matching images (solid lines) and non-matching images (dashed lines) individually. Panels on the right side **(B,D)** show the paired difference between matching and non-matching evaluations. In all panels the activation levels are obtained using Gabor filters (gray lines) and autoencoder filters (black lines). The errorbars in all panels are 95% confidence intervals.

The segmentation index increases over simulation iterations for matching and non-matching masks and images (Figure 12A). The matching conditions have a steeper ascent and reach a higher segmentation index compared to the non-matching conditions. The difference between the matching segmentation index and the non-matching segmentation index increases for both simulations using Gabor weights and autoencoder weights (Figure 12B). The simulations using regular Gabor receptive fields show larger differences between matching and non-matching segmentation indices compared to the autoencoder weights. The ratio between matching and non-matching segmentation indices is roughly the same for both types of receptive fields. This demonstrates systematic binding in the phase maps of matching segments.

An evaluation for different segment sizes individually reveals more differences between the Gabor and autoencoder features. The evaluations of the matching conditions show that the segmentation index increases for larger segments in the case of the autoencoder features but decreases for larger segments in the case of the Gabor features (Figure 12C). An explanation is that the autoencoder contains more features with low spatial frequencies while the Gabor features are restricted to one specific spatial frequency.

The paired difference between matching and non-matching evaluations shows that the Gabor filter and the autoencoder have roughly the same performance for large segment sizes (Figure 12D). For small segment sizes the autoencoder has a decreased segmentation performance. One possible explanation might be that the receptive field weights are not centered (compare Figure 3) and therefore different feature neurons might be slightly misaligned relative to the hand labeled segmentation masks, which are defined as polygons with arbitrary precision on the image.

Overall the results show a significant difference between the matching and the non-matching segmentation indices for all evaluated segment sizes. The paired difference between the matching and the non-matching conditions increases as the simulation of the randomly initialized phase variables slowly converges to a state with clusters in the circular phase space. After about 20 network iterations the paired difference in the segmentation index reaches a high plateau. Therefore, the coupled phase oscillator model achieves a stable segmentation of the natural image scenes with a coding of binding by synchrony.

#### 3.5.3. Segment boundaries

**Figure 13:**
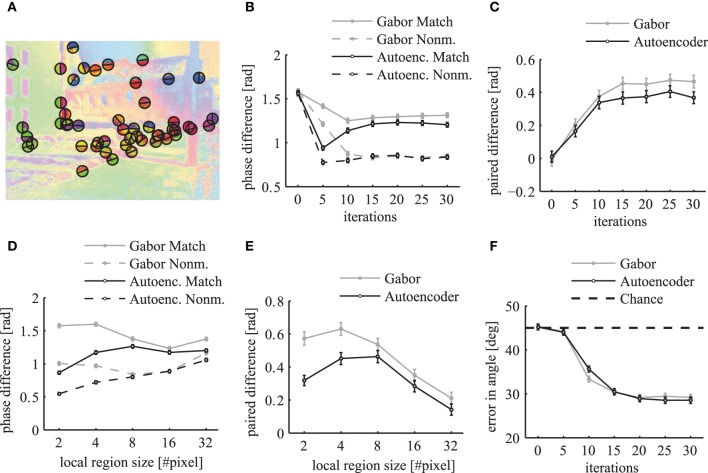
**Local evaluation of the phase segmentation**. Results were obtained using Gabor filters (gray) and autoencoder filters (black). **(A)** Illustration of the randomly selected locations on segment borders and the corresponding semicircles as described in the main text. **(B)** The local phase difference at random segment border locations of matching images (solid lines) and non-matching images (dashed lines). **(C)** The paired difference between the local phase differences evaluated on matching and non-matching images. **(D)** The mean local phase difference as a function of different sizes of the local circular regions over which the phase is evaluated. **(E)** The paired difference between matching and nonmatching images. **(F)** The mean error in the estimated angle of segment boundaries. All errorbars are 95% confidence intervals.

To evaluate how well the phase maps segment different labeled regions at their borders we calculate a metric at random locations of segment boundaries. We sample 50 random locations from all boundary lines of the segments in each simulated image from the LabelMe database. At these locations we use the angle of the segment boundary to divide a local region into two semicircles with a radius of 10 pixels such that one half lies approximately within the segment and the other half outside of the segment (Figure 13A). The mean phase difference between both semicircles decreases over simulation time (Figure 13B). The paired difference between the phase difference in matching compared to non-matching images shows that the phase difference over matching segment boundaries is significantly larger (Figure 13C).

The evaluation of the phase difference as a function of the size of this circular region shows that the segmentation performance using autoencoder features decreases for very small regions (Figures 13D,E). This might be due to the above described misalignments between the learned receptive field centers. For very large evaluation regions the performance decreases for both receptive field types because the circular regions are likely to extend beyond the hand labeled segment regions.

It is possible to evaluate the segmentation performance of the dynamic binding maps without the need for a baseline on non-matching images if we use an unbiased performance estimator with a clearly defined chance level. Therefore, we measure how well the phase map can predict the angle of the borders of segmentation masks. We use the phase variables at randomly sampled locations on segment boundaries (Figure 13A) and compute the image direction with the largest change in the phase variables. We define the local variance in phase at image position (*x*, *y*) as

(25) $$\begin{array}{l} \vartheta_{x,y}=1-\frac{1}{5\cdot K}\cdot |\sum_{k=1}^{K}e^{i\varphi_{x,y,k}}+e^{i\varphi_{x-1,y,k}}+e^{i\varphi_{x,y-1,k}}\\ \;+e^{i\varphi_{x+1,y,k}}+e^{i\varphi_{x,y+1,k}}| \end{array}$$

where the sum is over all *k* ∈ {1..*K*} feature maps. We use the structure tensor of the local variance in phase to estimate the principal directions. To compute the structure tensor we use a Gaussian window function with a standard deviation of 3 pixels and the second order central finite difference of the local variance in phase. The eigenvector of the structure tensor gives an estimate of the border direction of the segmentation mask. The evaluation of the phase maps shows that the mean error in the estimation of the boundary angles decreases over simulation time (Figure 13F). A minimum is reached after around 20 network iterations with an error of approximately 28° in comparison to the chance level of 45°. This demonstrates that the phase gradient systematically aligns itself orthogonal to the segment boundaries.

## 4. Discussion

Here we investigate the concept of binding by synchrony, as has been previously studied with abstract stimuli, in the context of unsupervised learning and natural stimuli. The model consists of coupled phase oscillators with a connectivity based on natural image statistics. Specifically, the correlation of neuronal activity governs the structure of local horizontal connections in the network. Hence the connections are not constructed according to a heuristic or intuition, but solely data driven. Therefore, we can expect it to generalize well to other cortical areas. We show that the sampled sparse connectivity based on positive correlations in induced activations by natural stimuli exhibits small-world properties. We hypothesize that the small world property is a signature of Gestalt laws in the form of regular local correlations (objects) that can be flexibly combined on a global scale. We show that these horizontal connections influence the dynamics of the phase variables such that an effective coding of contextual relationships between active neurons is implemented by phase synchronization. Therefore, our results reveal that the concept of binding by synchrony is viable for natural stimuli.

The evaluation of phase synchronization as a code for grouping and segmentation utilizes hand labeled image segments, corresponding to high level objects, as ground truth. The evaluations reveal that the phase maps are binding active neurons together if they encode different attributes of the same stimulus. It follows that the phase variables are coding global stimulus attributes in contrast to the coding of local stimulus attributes by the rate variables. The coding of these global contextual relationships is not directly influenced by the rate variables but only by their indirect modulation of the phase interactions. Furthermore, we illustrate that discontinuities are formed in the phase maps at the borders of segments and that these discontinuities can predict the orientation of segment boundaries. Therefore, our results suggest that the segmentation driven by bottom up dynamical processes using natural image statistics matches to a certain degree the top-down labeling of abstract image objects.

Our study connects three different subject areas: natural image statistics, dynamical models of neural networks and normative models of sensory processing. In the following we will discuss the motivations and implications of our study from each of these perspectives.

### 4.1. Choice of natural stimuli

The choice of “natural” stimulus material is not as obvious as it might seem. A more natural choice from a biological perspective would be to use stimulus material generated by a moving agent. For example videos from a camera mounted to a cat's head were used previously to analyze the spatio-temporal structure of natural stimuli (Kayser et al., 2003). A similar setup from a human perspective is also possible (Açik et al., 2009). But time variant stimuli require more computational resources and the high number of horizontal connections in our simulations is computationally expensive although it is implemented as a vectorized operation. In addition, the analysis of the phase segmentation maps would be more difficult in the case of moving stimuli because of the unknown time lag between stimulus onsets and the resulting dynamic phase maps. Therefore, we decided to not use videos as stimulus material in the present study.

Differences in eye movements given different stimulus classes might also play a role in shaping the statistics in the visual input received by the primary visual cortex. There might be important interactions between saccadic eye movements and the dynamics of the horizontal connections in the visual cortex. One could simulate saccadic movements on static images using saliency maps and use the resulting images for the feedforward processing in our model. But as with moving stimuli in general it would complicate the analysis and would not contribute directly to the understanding of the central questions of binding by synchrony.

The LabelMe database provides a large set of only static images. It has the advantage that the images are accompanied by labeled region masks of well defined objects. These high level labeled masks are often overlapping in the case of part-based segmentations of objects. The segmentation evaluation is tricky in the case of occluded objects. But the LabelMe database allows us to investigate the relationship between natural image statistics and the coding of high level image concepts. Therefore, we think it is a reasonable choice to use this database in our study.

### 4.2. Biological plausibility

As with most computational neural network models we have to ask ourselves in how far it is biologically plausible. To advance our knowledge about the underlying computation principles in the cortex, it is always a good choice to model only the level of detail which is necessary to explain the phenomena under investigation. Thereby we assure that the abstraction level of the model is as good as possible although it is very likely that some mechanisms below the level of detail modeled here play an important role in synchronization phenomena. We implement in our simulations the influence of correlated neuronal activity on large time scales to the network connectivity. Based on these connections we show how the dynamics on fast time scales can code for segmentation and binding. Therefore, we have to model the behavioral learning time scales (>days) to capture the natural image statistics and the dynamical network time scales (<seconds) simultaneously. Therefore, we consider the chosen network architecture of segregated rate and phase based coding suitable to investigate the role of correlated neuronal activity on the network dynamics and relational coding by synchronization.

The Kuramoto model restricts the dynamical interactions between coupled oscillators to a scalar phase variable. Breakspear et al. (2010) review this simplified model of coupled phase oscillators in the context of models of complex neurobiological systems. They find that it captures the core mechanisms of neuronal synchronization and a broad repertoire of rich, non-trivial cortical dynamics. Studies of the Kuramoto model mostly focus on regularly defined phase interactions without a separate network variable representing the activation levels of the oscillator neurons. This allows using mean-field approximations to further simplify the analysis of the Kuramoto model. In contrast, our study focuses on the simulation of heterogeneous connections which are modulated by heterogeneous activation levels induced by natural stimuli. Therefore, our simulation model is more similar to the diverse activations and connections found in biological neural systems but this comes with the drawback that a mean-field approximation is not warranted.

In principle two biological interpretations of the coupled phase oscillator model are possible. A conservative standpoint is an interpretation as a neural field model in which each network unit of our simulation represents a functional module, i.e., a cortical column, which is comprised of many biological neurons. In this case the phase variables would represent the average phase of a set of biological neurons, i.e., the phase of the local field potential. A second possible more fine-grained interpretation in which the phase oscillators represent individual biological neurons might seem far-fetched and oversimplified on first sight. Nonetheless the interpretation of the phase variables as spike timings might give further ideas about possible extensions of our proposed model. In this interpretation the oscillators represent the limit cycles of the dynamics of spike generation of biological neurons. The sinusoidal interaction function can then be related to an integral over the phase response function of a spiking neuron (Sturm and König, 2001). Furthermore, the spike interpretation could motivate the introduction of conduction delays in our model. This in turn might further allow studying spike-timing dependent plasticity in the context of a normative model.

Certainly, there are many phenomena that can only be modeled by more detailed spiking neuron models. For example spike-timing dependent plasticity could only be modeled with the phase oscillator model if we assume regular oscillatory firing but not in the case of irregular firing. For example, the ability of self-organizing recurrent networks (SORN) to learn spatio-temporal structures in the input depends on spike-timing dependent plasticity and irregular firing (Lazar et al., 2009). Similarly, Buonomano and Maass (2009) showed that spatiotemporal processing of natural stimuli can emerge from the dynamics of “hidden” neuronal states, such as short-term synaptic plasticity. Irregular firing is also needed for synfire chains of successively activated neural assemblies to explain the physiological measurements of spike patterns recurring with millisecond precision (Abeles, 1982). However, it might be possible to simulate some properties of synfire chains if we add more hierarchical layers and phase conduction in the feed forward projections in our model. Kumar et al. (2010) analyzed the coexistence of firing rate propagation and synchrony propagation in feed forward networks. Last but not least, self-organized criticality and cortical avalanches (Beggs and Plenz, 2003) can probably only be modeled with more detailed spike-based neuron models because the phenomenon requires a dynamical system of more complex coupled oscillators.

There are also other dynamical models of neural networks that were analyzed in the context of scene segmentation (Tononi et al., 1992). Wang and Terman (1997) described the local excitatory global inhibitory oscillator network (LEGION), which is comprised of units described by two differential equations that explicitly model a stable periodic orbit alternating between two phases with rapid transitions between them. This model has the advantage that fast synchronization of the coupled oscillators is possible. But it simulates each neuronal oscillation on a fast timescale and the synchronization of a population of neurons is only visible at certain simulated time points. In contrast, our phase model simplifies the phase plane to a continues phase variable averaged over many oscillatory periods, so that the phase relationships between all pairs of neurons is explicitly represented at all simulation time points. Another difference is that the implementation of LEGION involves many discontinuous operations to reduce the computation time. These discontinues operations prevent a normative model approach with optimizations using gradient descent. The full continuous dynamics in our model allows further optimizations of the horizontal connectivity using gradient descent methods.

In our model the forward connections are computed once and are then fixed during the phase simulation of horizontal connections. This is a very simplified model compared to the ongoing simultaneous processing of afferent and recurrent inputs in the cortex. But it is compatible with the fact that self sustained activity in the cortex can be measured also in the absence of stimulus inputs. Furthermore, computational models of cellular and network behavior support the conclusion that the cortical network operates in a recurrent rather than a purely feed-forward mode (Mariño et al., 2005). Therefore, it makes sense to simulate the lateral interactions decoupled from the time scale of forward projections that generate the activation levels.

We use the correlated neuronal activation levels as the probability to form horizontal intralayer connections. It was shown that the measured horizontal connectivity in the visual cortex of cats is indeed proportional to the correlation between receptive field wavelets in image statistics (Betsch et al., 2004). Our choice to use a sparse connectivity pattern instead of full connectivity with heterogenous connection strengths was initially intended as a computational shortcut to allow large-scale simulations. This sparse connectivity is in line with biological horizontal connectivity and reveals interesting properties that deserve further investigation. In the brain the binding of stimulus representations has to be distributed over many cortical areas. It was shown with graph theoretic measures that the sparse connectivity within the cortex is organized in hubs and shows properties of small-world networks (Sporns et al., 2004). One can speculate that this allows binding by temporal structure even between stimulus representations over distant cortical regions. Also in our network model the sampled sparse connection patterns generated from correlated neuronal activity were shown to have small-world properties in the case of synchronizing connections. Accordingly, we see in our network simulations fast synchronizations of distant neurons that are not directly connected. And in future studies our model could be extended to simulate even synchronizations between different cortical regions.

In the cortex a wide range of oscillatory frequencies at different spatial scales occur with cross-frequency couplings. This is highly prominent in different sleep stages (Belluscio et al., 2012) and plays an important role in memory encoding (Friese et al., 2012). Our model is highly simplified in the sense that all neurons are assumed to have the same oscillatory natural frequency. We simulate only horizontal connections between neurons with similar physiological properties which are operating in the same dynamical regime. In this context, the assumption that all active neurons are close to a similar dynamical limit cycle seems reasonable. In future work, several cortical rhythms could be implemented using several phase variables per neuron. One can conceive different algebraic structures which could efficiently represent cross-frequency couplings in the cortex. This would allow investigating fractal binding at different abstraction levels and segmentation at different scales.

In summary, the architecture of our model captures many important aspects of biological neural networks. In particular, it models the dynamical properties used for contextual coding and the unsupervised learning of statistics in natural stimuli. At the same time, our model keeps the simplicity required for the analysis of the network dynamics and allows relatively simple evaluations of the resulting phase relationships.

### 4.3. Comparison with other normative models

In recent years the abstraction from complex differential equations describing biological neural networks to normative models of rate-based sensory processing improved our knowledge on the underlying computational principles of the cortex (Olshausen and Field, 2005). Unsupervised learning of the inherent statistics in the sensory input seems to be one of the main mechanisms governing the structural connectivity between neurons in low level sensory areas of the cortex (Olshausen and Field, 1996; Wiskott and Sejnowski, 2002; Körding et al., 2004). On the other hand relatively few studies have investigated the relationship between unsupervised learning using correlated neuronal activity and the coding of contextual relationships through binding by synchrony. In this section we describe differences and similarities between our model and other normative models of sensory processing in the brain.

Wyss et al. (2006) and Franzius et al. (2007) show that rate-coding neurons form a hierarchy of processing stages resembling the ventral visual pathway. These studies use optimization functions of optimal stability and decorrelation while exposing the network to natural stimuli. Although these models provide important insights into the information processing mechanisms in the cortex, they don't take into account the processing of contextual information and lack an implementation of relational coding between different features. In a similar way to these studies, we use the statistics of natural stimuli not only to learn feature representations but also to explain relational coding in the context of binding by synchrony. This approach could allow combining multi-scale image segmentation and object recognition into a hierarchical neuronal network model. A prerequisite for analyzing the segmentation by synchrony in a hierarchical network is an unsupervised learning of the feed-forward connections to generate the activation levels for higher network layers. We have shown that the proposed segmentation by synchrony works with receptive fields obtained from convolutional autoencoders, which can be stacked to obtain the forward and backward connections within a hierarchy. This allows a completely unsupervised learning of feed-forward, feed-back and intralayer connections using natural image statistics. Binding and extraction of features can be accomplished simultaneously within the hierarchy.

Biologically inspired autoencoder models were shown to be efficient for unsupervised learning of receptive fields by minimizing the reconstruction error of the input (Coates et al., 2010). Complex valued autoencoders have similar to our model 2 variables per network node (Baldi and Lu, 2012). To our knowledge the available publications investigating complex valued autoencoders focus mainly on the aspect of learning compressed representations of complex valued inputs. They do not directly address the biological motivation of binding by synchrony. They are usually strictly defined on the typical complex algebra and are not described by a differential equation which corresponds to coupled oscillators. The formalism of complex valued autoencoders might be adapted to allow further abstractions of our model. This could support our understanding of the underlying computational principles of visual grouping and segmentation.

A very different and novel approach of coding contextual informations in autoencoder networks are mean-covariance restricted Boltzmann machines (Ranzato and Hinton, 2010). In these models latent hidden factors are used to efficiently represent the contextual information in the input in addition to the usual representation of pixel means in standard models of restricted Boltzmann machines. It was shown that the model can efficiently code pixel covariances in analogy to complex cells and pixel means in analogy to simple cells. However, the coding of contextual information in these models is limited to pair-wise interactions in the input layer. Therefore, this kind of generative model can capture only a linear combination of second order statistics so that contextual interactions between a large group of neurons is only possible through direct connections. In contrast, the grouping in our model is a dynamic process in which interactions between neurons are possible without a direct connection between them but through intermediate neurons. The reason is that our model uses a dynamical system approach with recurrent connections in contrast to probabilistic modeling of forward and backward connections.

Some mathematical theories of cortical processing mechanisms also take the contextual information into account. For example the free energy principle (Friston, 2010) and the theory of coherent infomax (Kay and Phillips, 2011) explicitly incorporate the context into single-variable local processors in the network. In contrast, the model presented in this paper takes the context into account in a separate phase variable, which codes relational properties similar to the dynamics on fast time scales in biological neural networks. Thereby our simulation allows to model higher order relational structures with a limited number of horizontal connections. In contrast, in the mathematical formalization of coherent infomax the contextual field input is assumed to be integrated into a single variable output of a local processor in the network. Thereby it doesn't allow implementing higher order relations between many local processors if the computational resources are limited. This limitation is of course only a matter of the used mathematical formalism and doesn't affect the general explanatory power of the free energy principle or the theory of coherent infomax. Therefore, in a broader sense our simulation model could be seen as an approximate implementation of these abstract concepts, although we use a biologically motivated architecture instead of a probabilistic derivation.

Our study combines aspects of these normative models of sensory processing and of detailed models of dynamical neural networks. We use only the statistics induced by natural images to learn unsupervised the forward and tangential phase connections. The supervised labeled segmentation masks are only used to evaluate how phase synchrony corresponds to a relational coding in the neural representation. Hence, the concept can be phrased completely in the form of a normative model. In future work, we plan to further formalize the model and conceive more complex learning rules for the phase interactions. These learning rules could replace the sampling of sparse connections from the correlation of activation by a more biologically motivated rule. For example, one could develop learning rules based on spike-timing dependent plasticity if phase delays are incorporated in the interactions of the network. This would additionally allow modeling phase locking between neurons and coding of syntactic relations in the network. These extensions to our model could provide new insights into the computational principles underlying higher order cognitive processes.

## 5. Conclusions

Our study revealed that the concept of binding by synchrony is viable in the context of unsupervised learning using natural stimuli. We show that the structural connectivity based on correlated activity leads to relational coding in a neural network model of coupled phase oscillators. The presented novel evaluation methodology for image segmentation revealed that the phase of neurons code global stimulus attributes. This strengthens the evidence that phase synchronization plays a key role to coordinate the spatially distributed information processing in the cortex. One could further speculate on how higher level coordination and binding between cortical areas might evolve from unsupervised learning based on correlated neuronal activity.

### Conflict of interest statement

The authors declare that the research was conducted in the absence of any commercial or financial relationships that could be construed as a potential conflict of interest.

## Footnotes

^1^http://www.di.ens.fr/~mschmidt/Software/minFunc.html
